# Microfluidic Synthesis of the Tumor Microenvironment-Responsive Nanosystem for Type-I Photodynamic Therapy

**DOI:** 10.3390/molecules27238386

**Published:** 2022-12-01

**Authors:** Chunyu Huang, Mingzhu Chen, Liang Du, Jingfeng Xiang, Dazhen Jiang, Wei Liu

**Affiliations:** 1 Key Laboratory of Artificial Micro and Nano-Structures of Ministry of Education, School of Physics and Technology, Wuhan University, Wuhan 430072, China; 2 Department of General Surgery & Guangdong Provincial Key Laboratory of Precision Medicine for Gastrointestinal Tumor, Nanfang Hospital, The First School of Clinical Medicine Southern Medical University, Guangzhou 510515, China; 3 Department of Radiation and Medical Oncology, Hubei Key Laboratory of Tumor Biological Behaviors, Hubei Cancer Clinical Study Center, Zhongnan Hospital of Wuhan University, Wuhan 430071, China; 4 School of Microelectronics, Wuhan University, Wuhan 430071, China; 5 Hubei Luojia Laboratory, Wuhan 430071, China

**Keywords:** microfluidics, aggregation-induced emission, drug delivery, photodynamic therapy

## Abstract

Type I photosensitizers with aggregation-induced emission luminogens (AIE-gens) have the ability to generate high levels of reactive oxygen species (ROS), which have a good application prospect in cancer photodynamic therapy (PDT). However, the encapsulation and delivery of AIE molecules are unsatisfactory and seriously affect the efficiency of a practical therapy. Faced with this issue, we synthesized the metal-organic framework (MOF) in one step using the microfluidic integration technology and encapsulated TBP-2 (an AIE molecule) into the MOF to obtain the composite nanomaterial ZT. Material characterization showed that the prepared ZT had stable physical and chemical properties and controllable size and morphology. After being endocytosed by tumor cells, ZT was degraded in response to the acidic tumor microenvironment (TME), and then TBP-2 molecules were released. After stimulation by low-power white light, a large amount of •OH and H_2_O_2_ was generated by TBP-2 through type I PDT, thereby achieving a tumor-killing effect. Further in vitro cell experiments showed good biocompatibility of the prepared ZT. To the best of our knowledge, this report is the first on the microfluidic synthesis of multifunctional MOF for type I PDT in response to the TME. Overall, the preparation of ZT by the microfluidic synthesis method provides new insight into cancer therapy.

## 1. Introduction

Health is the foundation of life, and cancer is among the main factors threatening people’s health and even lives [1,2,3]. The current mainstream methods in clinical treatment of cancer include surgery, chemotherapy, and radiotherapy [4]. However, the above traditional methods, used singly or in combination, exert serious side effects and cause certain risks to the human body during long-term treatment [5]. Therefore, there is an urgent need to explore safe and high-performance tumor ablation methods. In recent years, photodynamic therapy (PDT) has become a promising novel tumor treatment method owing to its apparent advantages, such as high spatial and temporal resolution and non-invasiveness [6]. Aggregation-induced emission luminogens (AIE-gens) have been studied as photosensitizers for the non-invasive treatment of cancer by photodynamic therapy (PDT) as they can generate the highly toxic reactive oxygen species (ROS) in response to light [7,8]. TBP-2, for example, is a typical AIE molecule that can realize type I PDT and produce hydroxyl radical (•OH) in the absence of oxygen in the tumor microenvironment (TME) [9]. As a result, these •OH radicals are substantially destructive to the vast majority of biological components, including tumor cells (e.g., DNA, protein, and the nucleus) [10,11]. However, type I PDT treatment could fully utilize the small amount of oxygen in hypoxic tumor tissue, which also leads to the better anti-hypoxic effect of type I PDT. Nonetheless, the efficient delivery of AIE molecules is a great challenge.

The metal-organic frameworks (MOFs), as a crystalline hybrid material, are formed by the coordination between metal ions, including transition metals such as Fe, Co, Ni, Cu, or lanthanide metals, and organic ligands, such as carboxylate, zolate, and phosphonate [12,13,14]. MOFs often exhibit excellent chemical and thermal stability and have the advantages of high porosity, regular pore structure, a large specific surface area, adjustable pore size, and diversity of topological structure [15]. With these advantages, MOF materials have attracted wide attention and show good application prospects in drug delivery and cancer therapy [16]. Among these MOFs, zeolitic imidazolate framework-8 (ZIF-8) has been the most extensively studied; they escort anti-tumor drugs owing to its high drug loading capacity, pH-responsive ability, and environmentally friendly synthesis [17]. For example, Li et al. encapsulated CO gas pro-drug manganese carbonyl (MnCO) and camptothecin (CPT) onto ZIF-8 MOF and designed a stable nano-drug delivery system (ZCM) for potent cancer therapy with good biocompatibility [18]. However, most ZIF-8 MOFs are chemically synthesized by traditional methods, which have low efficiency and require a longer time, which will limit their further application value.

For the development of nanomedicine and even clinical trials in the future, repeatable synthesis and mass preparation of nanomaterials are of great significance [19]. In the traditional chemical reactor, it is difficult to accurately realize the expected characteristics of nanomaterials due to the poor control of reaction kinetics [20,21]. As an ideal candidate, the precise control ability of the microfluidic chip provides a novel idea for the synthesis of nanomaterials (such as its higher cost-effectiveness and shorter production time). On microfluidic chips, large quantities of nanomaterials can be synthesized repeatedly [22]. More importantly, the synthesis based on microfluidics is conducted in a closed environment and is safe when dealing with toxic and flammable solvents [23]. For example, Hao et al. first designed a facile and straightforward flow synthesis strategy to control zinc oxide (ZnO) of various shapes [24]. The growth of ZnO can be well adjusted by varying substrate concentrations at different inlets and the flow rate of fluid; as a result, the shapes can be regulated to be short, rod, long rod, sphere, ellipsoid, cube, platelet, and urchin. The microfluidic method can provide a uniform environment for reactions and rapid mixing of substrates, and different microfluidic chips can be matched several times so that reaction conditions and a controllable reagent mixing mode are adjusted. The microfluidic chip has an infinite prospect for applications such as nanoparticle synthesis and drug delivery [25].

**Scheme 1:**
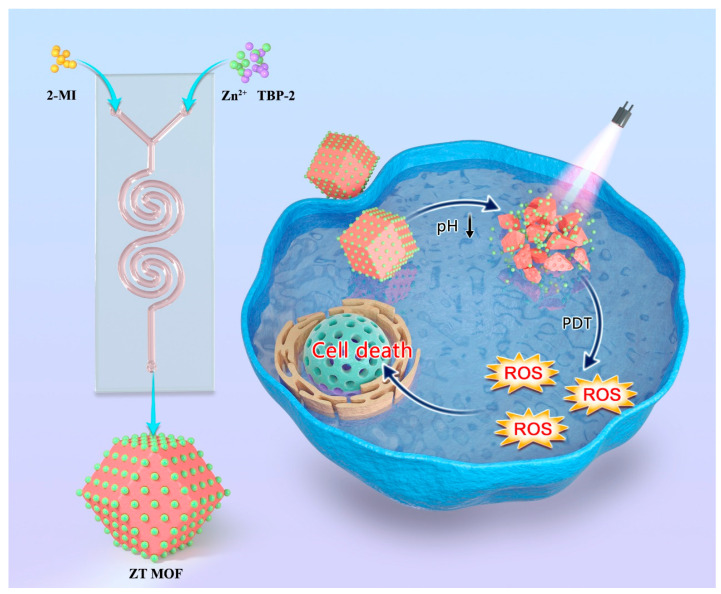
Schematic illustration of the microfluidic synthesis of a tumor microenvironment-responsive nanosystem for Type-I photodynamic therapy.

In this study, for enhanced PDT, we used a microfluidic synthesis method and attempted to design an intelligent ZIF-8@TBP-2 MOF (ZT) (Scheme 1). The microfluidic chip consists of two inlets, and raw materials react fully in a double-helix mixing channel in the chip and are output at a final outlet. The channel width was 1000 μm, and the height was 80 μm. The obtained ZT has good physicochemical properties and can respond well to the acidic TME to release TBP-2 after endocytosis by tumor cells, which can be subsequently used for tumor therapy. To the best of our knowledge, this is the first report of the microfluidic synthesis of a multifunctional MOF for type I PDT in response to the TME. Here, in vitro experiments demonstrated that the prepared MOF has low dark toxicity. This work can be used as a potential platform to sustainably synthesize nanoscale MOF and efficiently encapsulate AIE molecules for various healthcare applications.

## 2. Results and Discussion

**Figure 1:**
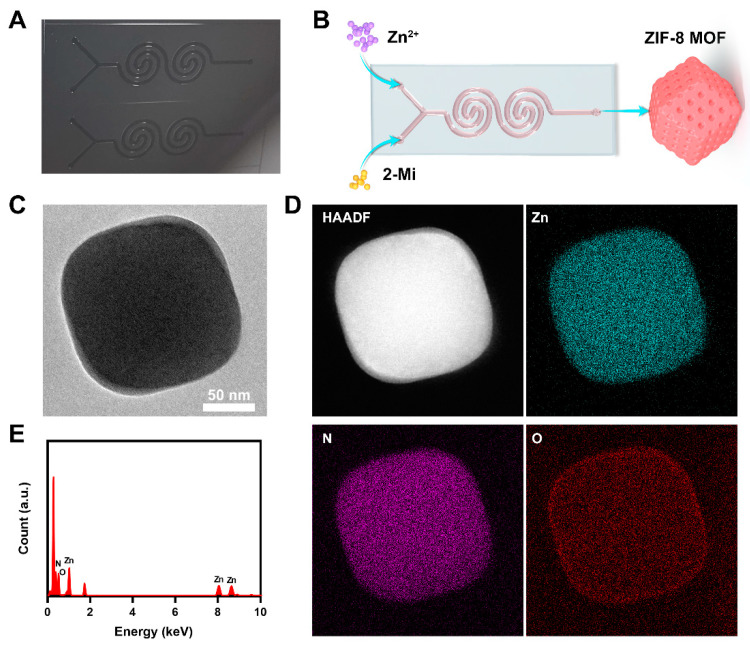
(**A**) SU-8 photoresist mold photograph. (**B**) The schematic model proposed for the synthesis of ZIF-8 MOF. (**C**) TEM image of the ZIF-8 MOF. (**D**) Mapping image of the ZIF-8 MOF. (**E**) EDX of ZIF-8 MOF.

Microfluidic technology, as one of the most promising technologies, can well integrate the nanoparticle synthesis platform into a small glass chip, rendering the synthesis of nanoparticles more controllable [26]. In general, the small size of nanoparticles, their large specific surface area, and their quantum size effect endow them with special properties lacking in conventional materials [27]. The mechanism for its formation is based on the classical nucleation theory; that is, the raw materials accumulate rapidly in the solution to form a specific supersaturated state, which overcomes the energy barrier for nucleation and finally leads to explosive nucleation [11,28]. The traditional synthesis method leads to uneven concentration of the system because it cannot produce uniform conditions in milliseconds or even seconds, which finally increases the variation between batches and reduces the repeatability [28,29]. Encouragingly, microfluidics can mix the base materials more effectively and produce a uniform state, thus stabilizing the physicochemical properties [30]. We designed a microfluidic chip with a two-spiral mixing channel to fabricate ZIF-8 MOFs. The transverse shear force in the double helix channel was more robust than that in the straight channel. This special double helix channel is advantageous to the mixing of the two raw materials. The spiral mixing channel makes for a more rapid and reasonable formation of ZIF-8 MOF. Figure 1A shows a photograph of the SU-8 photoresist mold. The channel was 1000 μm wide, with a height of 80 μm. Hence, such even and vigorous mixing leads to the generation of size-controlled small particles. The zinc nitrate solution (15 mg/mL of water) was passed through the first inlet, and the organic ligand molecule 2-methylimidazole (2-MI) (200 mg/mL of water) was passed through the second inlet (Figure 1B). The injection flow rate was 60 μL/min during the assembly of the ZIF-8 MOFs. As shown in Figure 1C, image obtained through transmission electron microscopy (TEM) determined that the diameter of the ZIF-8 MOF was about 110 nm. Figure 1D,E show typical elemental distribution mapping of ZIF-8 MOF: Zn, N, and O. ZIF-8 was prepared repeatedly, and its zeta potential was measured, which was +17.8, +16.8, and +18.4 eV, respectively. ZIF-8 prepared by microfluidic technology exhibited high reproducibility and may have better future application value than that prepared by the traditional hydrothermal method.

**Figure 2:**
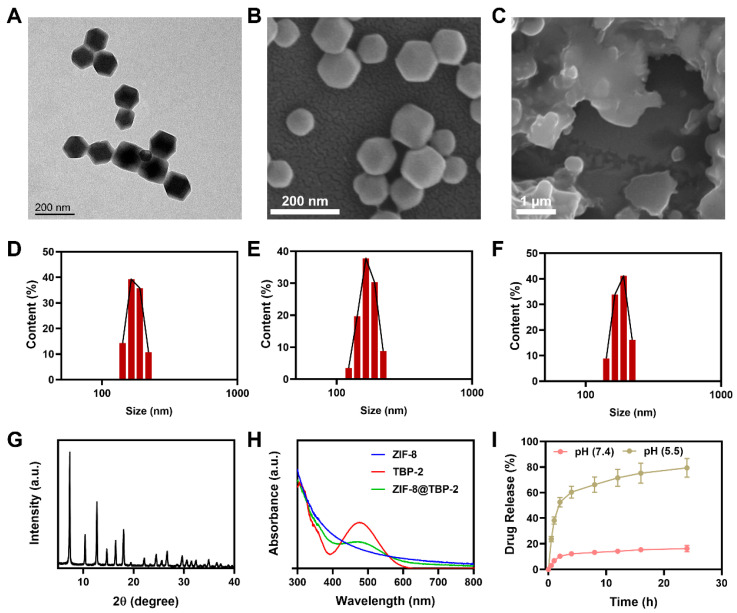
(**A**) TEM image of ZT. (**B**) SEM image of ZT. (**C**) SEM image of decomposed ZT. (**D**–**F**) Hydrodynamic diameter of ZT in PBS was assessed after 1, 2, and 3 days. (**G**) XRD spectra of ZIF-8. (**H**) UV-Vis spectra for ZIF-8, TBP-2, and ZIF-8@TBP-2 (ZT). (**I**) In vitro TBP-2 release profile at different pH levels from ZT.

Subsequently, we proceeded to prepare TBP-2-loaded ZIF-8 MOF (ZT). The TBP-2 molecules were mixed with Zn ions and allowed to flow in from channel 1, and 2-MI continued to flow in from channel 2. The chip structure and flow rate remained unchanged, and finally, uniform ZT nanomaterial was obtained at the exit. The results of TEM (Figure 2A) revealed no significant difference between the prepared ZT and ZIF-8 MOF in terms of morphology and size. We then verified the acid degradation properties of ZT. ZT was mixed with a neutral solution (pH 7.4) and a weak acid solution (pH 5.5) and placed aside for one day. Scanning electron microscopy (SEM) showed that ZT remained stable in a neutral environment without any change in structure (Figure 2B), while the typical octahedral structure of ZT in the acidic solution collapsed (Figure 2C). This can release the TBP-2 molecules that are loaded into ZT. Dynamic light scattering confirmed that the prepared ZT crystals were almost monodispersed; the hydrodynamic dimensions of ZT obtained through repeated preparation were ~176 ± 25 nm, ~171 ± 20 nm, and ~182 ± 28 nm (Figure 2D–F). Powder X-ray diffraction (XRD) showed that the ZIF-8 crystal pattern formed by the microfluidic method had obvious prominent peak positions (Figure 2G), including 011, 002, 112, 022, 013, and 222, confirming it to be a sodalite structure, which is the typical structure of ZIF-8. Furthermore, UV-Vis absorption spectra verified the successful synthesis of ZT (Figure 2H). Normal ZIF-8 exhibited no characteristic absorption peak, whereas ZIF-8@TBP-2 (ZT) exhibited the same absorption peak as the TBP-2 molecule; additionally, the encapsulation percentage of the ZIF-8 MOFs was approximately 48% for TBP-2. The efficiency of encapsulation of the conventional chemical synthesis method was only about 34% under the condition of a constant feeding ratio. Due to the pH response of ZIF-8 MOFs, we continued to test the drug release curve of ZT in different pH environments. ZT remained stable in a neutral solution, released only limited amounts of TBP-2, rapidly collapsed in a weak acid of pH 5.5, and released TBP-2 molecules (Figure 2I). Incubation of ZT with an acid solution for 4 h achieved approximately 60% drug release.

**Figure 3:**
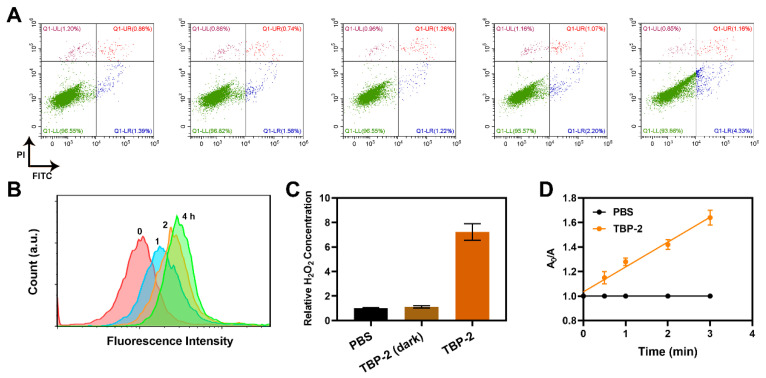
(**A**) Flow cytometry analysis of cell viability after treatment with different concentrations of ZIF-8 (0, 25, 50, 100, and 200 μg/mL). (**B**) Fluorescence intensity of cells co-incubated with ZT at different time points (0, 1, 2, and 4 h). (**C**) H_2_O_2_ generation in 4T1 cells upon white light irradiation with PBS and TBP-2. (**D**) TA decomposition rates induced by •OH generation associated with the indicated formulations under white light. (0.1 W/cm^2^, 5 min, TBP-2 was dissolved in a 1% DMSO solution).

TME often exhibits weak acidity, while material characterization verified that ZIF-8 MOF is structurally stable in a neutral aqueous solution but degrades in an acidic environment with a low pH. In addition, the high specific surface area of ZIF-8 and its adjustable porous structure make it an effective carrier for antitumor drugs (TBP-2 was encapsulated in this study). We first conducted flow cytometry to explore the biocompatibility of ZIF-8 prepared using a microfluidic chip. The results showed that ZIF-8 at high concentrations still had low biotoxicity (Figure 3A). After 4T1 cells were co-incubated with 200 μg/mL ZIF-8, less than 10% of cells were early apoptotic, late apoptotic, or necrotic. This is beneficial for long-term clinical applications. Due to the unique fluorescence characteristics of TBP-2, the content of ZT in 4T1 cells at different time points was analyzed by flow cytometry. The amount of ZT entering cells within 4 h was positively correlated with time (Figure 3B). We continued to measure the H_2_O_2_-production capacity of TBP-2 molecules and compared with the PBS-treated group and the TBP-2 (dark) group. TBP-2 could produce a large amount of H_2_O_2_ after light exposure (Figure 3C). We carried out a TA degradation test to verify the •OH generating potential of TBP-2 and observed that TBP-2 could induce the production of •OH under light stimulation (Figure 3D). ROS are the main molecules produced by the body during oxidative stress and are important factors in the development and recurrence of tumorigenesis. However, recent studies have revealed that ROS can achieve the treatment goal by accelerating tumor cell death. As a kind of ROS, •OH kills cells by damaging intracellular DNA, lysosomes, mitochondria, etc., to promote tumor cell apoptosis and play an anti-tumor role [31]. TBP-2 can directly respond to light to produce •OH, which possibly combines with MOF to achieve a good anti-tumor effect (Figure S1, see the Supplementary Materials).

**Figure 4:**
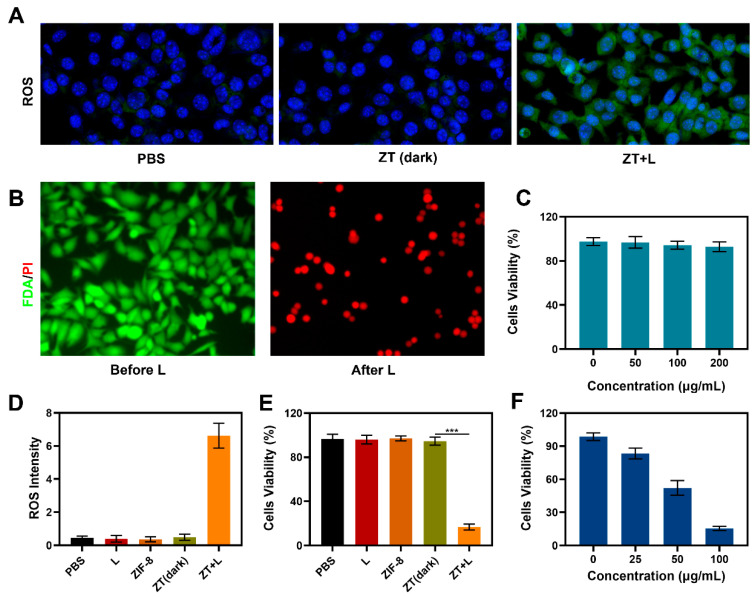
(**A**) Measurement of tumor cell ROS fluorescence after the different treatments. (**B**) Fluorescence images of the 4T1 cells after the different treatments. Live cells are shown in green and dead cells are shown in red. (**C**) Cell viability after treatment with different concentrations of ZT (dark condition) (0, 50, 100, and 200 μg/mL). (**D**) Quantitative analysis of ROS production in (**A**). (**E**) Cell viability after indicated treatment. *** *p* < 0.005; Student’s *t*-test. (**F**) Cell viability after treatment with different concentrations of ZT under white light.

Considering the above results, we continued to promote in vitro cell experiments. First, the 2′,7′-Dichlorofluorescin diacetate (DCFH-DA) kit was used to verify the ROS production ability of each group. The DCFH-DA itself has no fluorescence and is hydrolyzed to produce DCFH after entry into the cells, while intracellular ROS can oxidize DCFH to DCF, which fluoresces green light. The intensity of green fluorescence is directly proportional to the level of ROS [32]. As shown in Figure 4A, both the conventional control group and the pure material group showed a dim green fluorescence signal, while the ZT + L group produced good fluorescence intensity, indicating its strong ROS generation ability (Figure 4D). We also verified a positive correlation between ROS production and ZT concentration (Figure 4C). We then performed a plate clone formation assay to examine the ability of ZT + L to inhibit cell proliferation and observed that neither the ZT group nor the L group had any effect on cell proliferation, and after several generations of proliferation, the cells formed a typical population. However, ZT + L combined treatment significantly inhibited cell proliferation. Subsequently, we continued the live-dead cell staining assay and observed that the ZT + L group hardly exhibited green fluorescence (FDA) but had strong red fluorescence (DI), indicating high cytotoxicity in the ZT + L group (Figure 4B). Similarly, the MTT assay confirmed that the ZT + L group had the lowest cell viability when compared to the other control groups (Figure 4E). We verified the cell viability after treatment with different concentrations of ZT using an MTT kit and observed a negative correlation between cell viability and the concentration of ZT (Figure 4F). In conclusion, ZT prepared by a microfluidic platform can cooperate with L to produce a large amount of ROS, amplify oxidative stress, and thus induce high phototoxicity.

## 3. Conclusions

In conclusion, we explored a microfluidic method for the rapid fabrication of ZT composite nanomaterials responsive to TME through a one-step integration process for efficient type I PDT. ZT prepared by a microfluidic technology could effectively overcome the encapsulation problem of AIE molecules and exhibited good physical and chemical properties as well as biocompatibility. After reaching tumor cells, ZT degrades and releases TBP-2 in response to acidic TME and cooperates with white light irradiation to achieve efficient ROS production and phototoxicity, thereby killing tumor cells. This is the first report on the use of microfluidic chips for type I PDT to provide a potential approach for future biological applications of AIE molecules. At present, the application of nanomaterials to clinical practice is still a huge challenge, and the traditional transformation efficiency and repeatability are low. Microfluidic technology can design new platforms that are easy to use, making them more cost-effective and conducive to the production of high-repeatability and stable nanomaterials, which are expected to be used for further clinical research and industrial-scale production. We will continue to study the feasibility of nanomaterials prepared by microfluidic chips and attempt the synthesis and subsequent application of various novel nanosystems.

## Data Availability

Not applicable.

## Supplementary Materials

The following supporting information can be downloaded at: https://www.mdpi.com/article/10.3390/molecules27238386/s1.

## Sample Availability

Not available.

## References

[1] Ho D. (2020). Artificial intelligence in cancer therapy. Science.

[2] Ai K., Huang J., Xiao Z., Yang Y., Bai Y., Peng J. (2021). Localized surface plasmon resonance properties and biomedical applications of copper selenide nanomaterials. Mater. Today Chem..

[3] Zhu Y., Shi H., Li T., Yu J., Guo Z., Cheng J., Liu Y. (2020). A Dual Functional Nanoreactor for Synergistic Starvation and Photodynamic Therapy. ACS Appl. Mater. Interfaces.

[4] Liu R., Gong L., Zhu X., Zhu S., Wu X., Xue T., Yan L., Du J., Gu Z. (2022). Transformable Gallium-Based Liquid Metal Nanoparticles for Tumor Radiotherapy Sensitization. Adv. Healthc. Mater..

[5] Wang Z., Shao D., Chang Z., Lu M., Wang Y., Yue J., Yang D., Li M., Xu Q., Dong W.F. (2017). Janus Gold Nanoplatform for Synergetic Chemoradiotherapy and Computed Tomography Imaging of Hepatocellular Carcinoma. ACS Nano.

[6] Zhu D., Zhang J., Luo G., Duo Y., Tang B.Z. (2021). Bright Bacterium for Hypoxia-Tolerant Photodynamic Therapy Against Orthotopic Colon Tumors by an Interventional Method. Adv. Sci..

[7] Zhang T., Zhang J., Wang F.B., Cao H., Zhu D., Chen X., Xu C., Yang X., Huang W., Wang Z. (2022). Mitochondria-Targeting Phototheranostics by Aggregation-Induced NIR-II Emission Luminogens: Modulating Intramolecular Motion by Electron Acceptor Engineering for Multi-Modal Synergistic Therapy. Adv. Funct. Mater..

[8] Li Y., Wu Q., Kang M., Song N., Wang D., Tang B.Z. (2020). Boosting the photodynamic therapy efficiency by using stimuli-responsive and AIE-featured nanoparticles. Biomaterials.

[9] Zhu D., Zhang T., Li Y., Huang C., Suo M., Xia L., Xu Y., Li G., Tang B.Z. (2022). Tumor-derived exosomes co-delivering aggregation-induced emission luminogens and proton pump inhibitors for tumor glutamine starvation therapy and enhanced type-I photodynamic therapy. Biomaterials.

[10] Zhu-Ge X., Xi D.-M., Zhang S.-S. (2022). Multimodal tumor therapy based on chemodynamic therapy. Chin. J. Anal. Chem..

[11] Zhao T., Wu W., Sui L., Huang Q., Nan Y., Liu J., Ai K. (2022). Reactive oxygen species-based nanomaterials for the treatment of myocardial ischemia reperfusion injuries. Bioact. Mater..

[12] Zhao Q., Gong Z., Li Z., Wang J., Zhang J., Zhao Z., Zhang P., Zheng S., Miron R.J., Yuan Q. (2021). Target Reprogramming Lysosomes of CD8+ T Cells by a Mineralized Metal-Organic Framework for Cancer Immunotherapy. Adv. Mater..

[13] Su L., Wu Q., Tan L., Huang Z., Fu C., Ren X., Xia N., Chen Z., Ma X., Lan X. (2019). High Biocompatible ZIF-8 Coated by ZrO2 for Chemo-microwave Thermal Tumor Synergistic Therapy. ACS Appl. Mater. Interfaces.

[14] Jiang W., Zhang H., Wu J., Zhai G., Li Z., Luan Y., Garg S. (2018). CuS@MOF-Based Well-Designed Quercetin Delivery System for Chemo-Photothermal Therapy. ACS Appl. Mater. Interfaces.

[15] Huang J., Xu Z., Jiang Y., Law W.C., Dong B., Zeng X., Ma M., Xu G., Zou J., Yang C. (2021). Metal organic framework-coated gold nanorod as an on-demand drug delivery platform for chemo-photothermal cancer therapy. J. Nanobiotechnol..

[16] Ren S.Z., Wang B., Zhu X.H., Zhu D., Liu M., Li S.K., Yang Y.S., Wang Z.C., Zhu H.L. (2020). Oxygen Self-Sufficient Core-Shell Metal-Organic Framework-Based Smart Nanoplatform for Enhanced Synergistic Chemotherapy and Photodynamic Therapy. ACS Appl. Mater. Interfaces.

[17] Geng S., Lou R., Yin Q., Li S., Yang R., Zhou J. (2021). Reshaping the tumor microenvironment for increasing the distribution of glucose oxidase in tumor and inhibiting metastasis. J. Mater. Chem. B.

[18] Li Y., Liu Z., Zeng W., Wang Z., Liu C., Zeng N., Zhong K., Jiang D., Wu Y. (2021). A Novel H2O2 Generator for Tumor Chemotherapy-Enhanced CO Gas Therapy. Front. Oncol..

[19] Balachandran Y.L., Li X., Jiang X. (2021). Integrated Microfluidic Synthesis of Aptamer Functionalized Biozeolitic Imidazolate Framework (BioZIF-8) Targeting Lymph Node and Tumor. Nano Lett..

[20] Wang J., Sui L., Huang J., Miao L., Nie Y., Wang K., Yang Z., Huang Q., Gong X., Nan Y. (2021). MoS2-based nanocomposites for cancer diagnosis and therapy. Bioact. Mater..

[21] Huang J., Huang Q., Liu M., Chen Q., Ai K. (2022). Emerging Bismuth Chalcogenides Based Nanodrugs for Cancer Radiotherapy. Front. Pharmacol..

[22] Lengert E.V., Trushina D.B., Soldatov M., Ermakov A.V. (2022). Microfluidic Synthesis and Analysis of Bioinspired Structures Based on CaCO3 for Potential Applications as Drug Delivery Carriers. Pharmaceutics.

[23] Cai Q., Castagnola V., Boselli L., Moura A., Lopez H., Zhang W., de Araujo J.M., Dawson K.A. (2022). A microfluidic approach for synthesis and kinetic profiling of branched gold nanostructures. Nanoscale Horiz..

[24] Hao N., Xu Z., Nie Y., Jin C., Closson A.B., Zhang M., Zhang J.X.J. (2019). Microfluidics-enabled rational design of ZnO micro-/nanoparticles with enhanced photocatalysis, cytotoxicity, and piezoelectric properties. Chem. Eng. J..

[25] Wang J., Ma P., Kim D.H., Liu B.F., Demirci U. (2021). Towards Microfluidic-Based Exosome Isolation and Detection for Tumor Therapy. Nano Today.

[26] Cheng R., Jiang L., Gao H., Liu Z., Makila E., Wang S., Saiding Q., Xiang L., Tang X., Shi M. (2022). A pH-Responsive Cluster Metal-organic Framework Nanoparticle for Enhanced Tumor Accumulation and Antitumor Effect. Adv. Mater..

[27] Liu Y., Luo J., Liu Y., Liu W., Yu G., Huang Y., Yang Y., Chen X., Chen T. (2022). Brain-Targeted Biomimetic Nanodecoys with Neuroprotective Effects for Precise Therapy of Parkinson’s Disease. ACS Cent. Sci..

[28] Zhu D., Chen H., Huang C., Li G., Wang X., Jiang W., Fan K. (2022). H2O2 Self-Producing Single-Atom Nanozyme Hydrogels as Light-Controlled Oxidative Stress Amplifier for Enhanced Synergistic Therapy by Transforming “Cold” Tumors. Adv. Funct. Mater..

[29] Liu Y., Luo J., Chen X., Liu W., Chen T. (2019). Cell Membrane Coating Technology: A Promising Strategy for Biomedical Applications. Nano-Micro Lett..

[30] Yang R., Ouyang Z., Guo H., Qu J., Xia J., Shen M., Shi X. (2022). Microfluidic synthesis of intelligent nanoclusters of ultrasmall iron oxide nanoparticles with improved tumor microenvironment regulation for dynamic MR imaging-guided tumor photothermo-chemo-chemodynamic therapy. Nano Today.

[31] Zhu D., Ling R., Chen H., Lyu M., Qian H., Wu K., Li G., Wang X. (2022). Biomimetic copper single-atom nanozyme system for self-enhanced nanocatalytic tumor therapy. Nano Res..

[32] Ye P., Li F., Zou J., Luo Y., Wang S., Lu G., Zhang F., Chen C., Long J., Jia R. (2022). In Situ Generation of Gold Nanoparticles on Bacteria-Derived Magnetosomes for Imaging-Guided Starving/Chemodynamic/Photothermal Synergistic Therapy against Cancer. Adv. Funct. Mater..

