# Supplementary material for: Microfluidic Synthesis of the Tumor Microenvironment-Responsive Nanosystem for Type-I Photodynamic Therapy
Source: Molecules. 2022 Dec 1;27(23):8386. doi: 10.3390/molecules27238386 (PMC9736763; doi:10.3390/molecules27238386)
Supplement: Supplementary file 1 [file molecules-27-08386-s001.zip › molecules-2019264-supplementary.pdf]

Experimental Procedures

Materials and reagents.

Hydrogen peroxide (H_2O_2) fluorescence detection kit, p-phthalic acid (TA) was purchased from Sigma-Aldrich. Dichlorofluorescin diacetate (DCFH-DA) were obtained from Solarbio life sciences. All of the aqueous solutions were prepared using purified deionized (DI) water purified with a purification system (Direct-Q3, Millipore, USA). The other solvents used in this work were purchased from Sinopharm Chemical Reagent (China) and Aladdin-Reagent (China).

Cell culture

4T1 breast cancer cell line was obtained from the Cell Bank of the Chinese Academy of Sciences and incubated in RPMI-1640 medium supplemented with 10% FBS in a humidified atmosphere at 37°C.

Synthesis of TBP-2 AIEgens

Into a 100 mL two-necked round bottom flask equipped with a condenser, was dissolved TBP (200 mg, 0.44 mmol) in 10 mL acetonitrile. (3-Bromopropyl) trimethylammonium bromide (170 mg, 0.66 mmol) was then added and the mixture was heated to reflux for 8 h. After cooling to room temperature, the mixture was poured into diethyl ether. The dark red precipitates formed were filtered by suction filtration and purified by flash column chromatography (DCM/MeOD = 5:1) to afford the CCSired product (200 mg, 65%). 1H NMR (400 MHz, MeOD-d4), δ (TMS, ppm): 9.17-9.15 (2H, d), 9.07-9.05 (2H, d), 8.52-8.50 (1H, d), 8.10-8.07 (3H, dd), 7.39-7.35 (4H, t), 7.20-7.13 (8H, m), 4.83-4.80 (2H, t), 3.67-3.63 (2H, m), 3.26 (9H, s), 2.69 (2H, m). 13C NMR (400 MHz, MeOD-d4), δ (TMS, ppm): 153.86, 153.52, 152.78, 149.33, 147.16, 144.25, 138.01, 132.28, 130.37, 129.24, 126.63, 126.28, 125.05, 123.74, 121.49, 62.49, 57.10, 52.60, 24.73. HRMS (TOF-MS), m/z: calcd. for C31H25N4S2+: 278.6301, found: 278.6292 [M-2Br]2+.

Design and fabrication of the microfluidic chip

An Auto CAD software was used to design a gradually widening S-shaped microfluidic channel, which includes two inlets, a two-spiral mixing spiral channel and one outlet for collecting the product. The channels were all 1000 μm in width and 80 μm in height. The spiral channels first rotated for loops in the counterclockwise direction. After an S-shaped junction, the spiral channels changed the counterclockwise direction for clockwise direction and rotated for another two loops. The transparent microfluidic channel plate was obtained by casting PDMS on the microfluidic channel mold. The microfluidic chip was fabricated using plasma bonding technology.

Preparation and characterization of ZIF-8 MOF and ZT

Zinc nitrate solution (15 mg/ mL) and 2-methylimidazole (200 mg/ mL) was dissolved in deionized water. The zinc solution and different ligand solutions were injected into the microfluidic channels by syringe pumps to prepare ZIF-8 and ZT. The morphology structures of ZIF-8 and ZT (pure condition and acid condition) nanoparticles were observed by the TEM (JEOL-2100) and SEM. The HAADF TEM images were obtained in JEM-F200 transmission electron microscope. Uv-vis spectra of different samples were recorded by the Uv-vis spectrophotometry Lambda 35 (Perkin-Elmer). Hydrodynamic diameter and zeta potential were detected by the dynamic light scattering (Nano-ZS ZEN3600).

Hydroxyl radicals (•OH) Generation Detection of TBP-2

TA was used as the •OH monitoring agent. In the experiments, 300 μL of TA solution was added to 2 mL of TBP-2 suspension or PBS solution and white light (WL, 0.1W/cm^2, 5 min) was employed as the radiation source. The absorption of TA at 425 nm was recorded at various irradiation times to obtain the decay rate. The •OH production was also assessed using TMB as the substrate.

Hydrogen peroxide (H_2O_2) detection *in vitro*

4T1 cells (8×10^4 per plate) were incubated with two different group: (1) PBS+ white light irradiation (L); (2) TBP-2 and (3) TBP-2+L. The TBP-2 concentration was 10 μg/mL. Then, cells were exposed to white laser radiation (0.1W/cm^2) for 5 min. After 4 h's culturing, the cells were collected by centrifugation (225 g, 3 min), followed by dispersion in acetone (1 mL for 5×10^6 cells) and sonication for 10 min in an ice bath. Afterward, the suspension was centrifuged (8000 g, 10 min) to collect the supernatant. The H_2O_2 content was assessed by the commercial H_2O_2 assay kit based on the product protocol.

Fluorescence Imaging

For determination of ROS levels via fluorescent imaging, CT26 cells were incubated for 6 h in five different groups: (1) PBS; (2) white laser irradiation (L, 0.1 W/cm^2); (3) ZIF-8; (4) ZT and (5) ZT + L. The ZIF-8 concentration was 200 μg/mL in group 3, 4 and 5. Then, cells in group 2 and 5 were exposed to white laser radiation (0.1 W/cm^2) for 10 min. Then, the fluorescent dye, DCFH-DA (10 μmol/L), was added and co-incubated at 37 °C. ROS level was determined by fluorescence microscopy.

***In vitro* anticancer effect of ZT**

The anticancer effect was measured by MTT assay. 4T1 cells (8×10^4 per plate) were incubated with five different group: (1) PBS; (2) white laser irradiation (L, 0.1 W/cm^2); (3) ZIF-8; (4) ZT and (5) ZT + L. The ZIF-8 concentration was 200 μg/mL in group 3, 4 and 5. Then, cells in group 2 and 5 were exposed to white laser radiation (0.1 W/cm^2) for 10 min. At the end of the incubation, 5 mg/mL MTT PBS solution was added, and the plate was incubated for another 4 h. Finally, the absorbance values of the cells were determined by using a microplate reader (Emax Precision, USA) at 570 nm. The background absorbance of the well plate was measured and subtracted. The cytotoxicity was calculated by dividing the optical density (OD) values of treated groups (T) by the OD values of the control (C) (T/C × 100%). Cell**s** viability of 4T1 cells after ZT+L treatment with different concentration was conducted in a similar way.

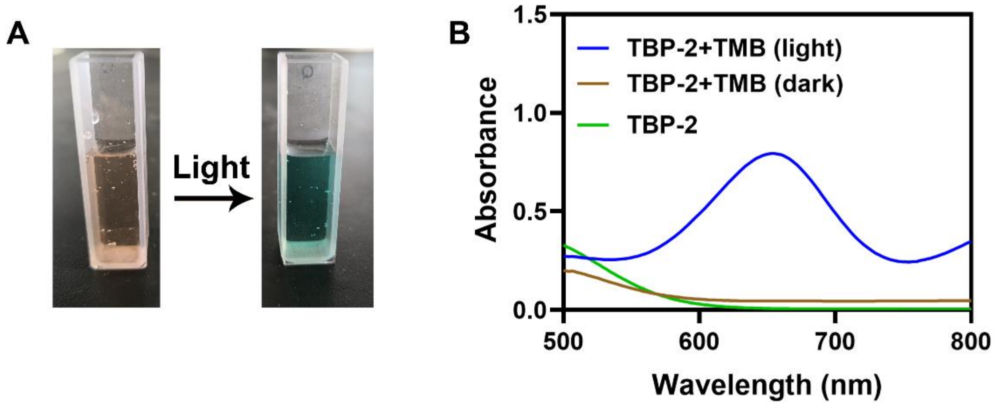

Figure S1. (A) Color change of mixed liquid (TMB + TBP-2) before and after light irradiation. (A) UV−vis absorbance spectra changes of TMB in different reaction systems.
